# Special Hospitals and Their Visiting Staffs

**Published:** 1907-06-15

**Authors:** 


### Special Hospitals and their Visiting Staffs.

Mr. Francis Roe, of Old Street, Lecturer in First Aid to the London County Council, gives a startling account of the conduct of "a member of the visiting staff of a hospital for diseases of the throat, nose, and ear in London, whose consulting rooms are in the vicinity of Cavendish Square." It behoves the members of the Medical Staff at every special Throat hospital in London to meet at once, and either to refute the statements made by Mr. Francis Roe, or to call for the resignation of any member of the visiting staff should it be proved that he has acted in the manner described.

In substance, Mr. Roe states that a lady in poor circumstances was forced to take her son to the Throat Hospital. There he was seen by one of the visiting staff who advised an operation and fixed the patient's next visit to the hospital in a fortnight. In the interval the practitioner in question wrote from his private address to the mother of the patient referring to the case seen at the hospital, and invited the mother to bring the boy to his consulting room. The mother went and was then told that the operation would have to be very carefully performed, that the boy was too delicate to be taken home after it, and that hæmorrhage might occur after they got home where there would be no one to attend to it. The practitioner suggested the admission of the boy to a nursing home, two or three of which he mentioned, where the operation could be performed and he might be sent for immediately in case of necessity. When the lady asked the probable cost, a reduced fee was first suggested, and then, on the mother stating her total inability to pay any fee, the consultant suggested that the mother should wait till her financial position improved sufficiently to have the operation done privately as he proposed. The mother, with Mr. Roe's assistance, then sent the patient to the Throat Surgeon at a general hospital where the necessary operation has been performed.

Mr. Francis Roe defines this extraordinary case as another form of hospital abuse. He adds "I do not know whether this is the usual method adopted by members of the staffs of the special hospitals to increase their private practice; but if it is so, that may be the explanation of the fact that not only are the necessitous poor (who, by the way, are always asked to pay something) treated at these institutions but nobody who will pay whatever he or she is disposed to is refused admission. I have heard people in comfortable circumstances boast that they need not pay doctors, as all they had to do was to go to a hospital and give a couple of shillings, and thus have the advantage of consulting West End specialists." We agree with Mr. Francis Roe that this is a subject and charge which needs immediate investigation, for "if a consultant uses his position on the staff of a hospital as a vantage point from which to tout for private patients, he should be brought to book, just as much as the general practitioner who touts by means of a friendly society." Special hospitals have greatly improved in many respects of late years, and they owe it to their present position to protect themselves from the possibility of the recurrence of any such case as that just described. Many members of the visiting staffs of the London special hospitals are among the best qualified and most honourable men in the profession. We confidently look to them to take prompt action in this matter, and to provide a remedy and a safeguard without a moment's avoidable delay.

